# Isolation and Characterization of a Low-Temperature, Cellulose-Degrading Microbial Consortium from Northeastern China

**DOI:** 10.3390/microorganisms12061059

**Published:** 2024-05-24

**Authors:** Jiaoyang Ji, Maia Escobar, Shijia Cui, Wei Zhang, Changjie Bao, Xuhan Su, Gang Wang, Sitong Zhang, Huan Chen, Guang Chen

**Affiliations:** 1 College of Life Science, Jilin Agricultural University, Changchun 130022, China; 2 Key Laboratory of Straw Comprehensive Utilization and Black Soil Conservation, Ministry of Education, Jilin Agricultural University, Changchun 130118, China; 3 Jilin Province Hydraulic Research Institute, Changchun 130022, China

**Keywords:** cold-adapted microorganisms, corn straw degradation, microorganism product, soil microorganisms

## Abstract

The lack of efficient ways to dispose of lignocellulosic agricultural residues is a serious environmental issue. Low temperatures greatly impact the ability of organisms to degrade these wastes and convert them into nutrients. Here, we report the isolation and genomic characterization of a microbial consortium capable of degrading corn straw at low temperatures. The microorganisms isolated showed fast cellulose-degrading capabilities, as confirmed by scanning electron microscopy and the weight loss in corn straw. Bacteria in the consortium behaved as three diverse and functionally distinct populations, while fungi behaved as a single population in both diversity and functions overtime. The bacterial genus *Pseudomonas* and the fungal genus *Thermoascus* had prominent roles in the microbial consortium, showing significant lignocellulose waste-degrading functions. Bacteria and fungi present in the consortium contained high relative abundance of genes for membrane components, with amino acid breakdown and carbohydrate degradation being the most important metabolic pathways for bacteria, while fungi contained more genes involved in energy use, carbohydrate degradation, lipid and fatty acid decomposition, and biosynthesis.

## 1. Introduction

Lignocellulosic agricultural residues, such as corn straw, are valuable industrial resources for the production of biofuels, polymers, and chemicals. The accumulation and waste of this potentially useful resource is both an environmental and industrial issue that needs to be tackled [1]. Only 10% of corn straw residues are returned to the fields in the northeast region of China, the main area of corn production, due to low temperatures and high winds that reduce the activity of microorganisms and negatively affect straw degradation [2]. Therefore, special attention has been given to the isolation and characterization of cellulose-degrading microorganisms that are active at low temperatures [3].

In industrial processes, it is often essential to pre-treat the raw straw residues [4,5]. There are various methods to achieve this, e.g., chemical methods (acid, alkaline wet oxidation, organosolv, and ammonia fiber explosion), mechanical strategies (comminution, steam explosion, microwaves, and radio-frequency heating), biological treatments, or a combination of these approaches [6]. Chemical pre-treatment can produce secondary pollution, while the other methods are energy-intensive. Biological pre-treatment seems to be the best option because it does not have the disadvantages of chemical pre-treatment and has relatively low cost [7].

Lignocellulosic agricultural residues are mainly composed of the plant cell-wall components, including cellulose, hemicellulose, and lignin. These materials contain carbon biomass at high levels, which the cellulose-degrading microorganisms can utilize [8]. However, cellulose has a crystalline structure that makes it very difficult to degrade, and its interactions with hemicellulose and lignin add an extra protective layer [7,9]. The molecular composition of hemicellulose is largely varied, including xylan, mannan, galactan, and arabinan polymers; therefore, a large number of enzymes are required for its efficient degradation [10]. Three types of enzymes acting synergistically are necessary for the degradation of cellulose: i.e., cellobiohydrolases (CBHs) attack the ends of the molecule, producing cellobiose; glycosidases (BGls) convert the cellobiose to glucose monomers; and endoglucanases (EGs) act in the middle of cellulose chains, reducing the degree of polymerization [11]. In addition, other enzymes, including glycoside hydrolases from family 61, expansins, and swollenin, can contribute to the degradation process, along with some proteins without cellulose-degrading abilities themselves [10]. Lignin degradation depends on the cooperation of three enzymes, including laccase, lignin peroxidase, and manganese peroxidase [7], showing varied ways for microorganisms to degrade cellulolytic materials. In general, they can be classified either as free or in a complex, which includes several cellulolytic enzymes bound to a scaffold protein (cellulosome) [10,12].

Soil microorganisms play key roles in the recycling of organic matter and maintaining the Earth’s geochemical cycles by degrading cellulose and other components of straw residues into fermentable sugars [13]. Many microorganisms have been isolated from soils and used in the degradation of corn straw, including cellulose-degrading bacteria *Bacillus subtilis* [14], *Lotharella globose* and *Trichosporon loubieri* [3], and *Klebsiella* [15], while many studies have also explored the thermophilic microorganisms [16,17]. Although there are reports of low-temperature cellulases, little work has been conducted on corn straw degradation at low temperatures [3]. Therefore, many low-temperature, straw-degrading microorganisms remain undiscovered, and their specific modes of action are unknown. However, it has been shown that temperature can affect the structure of straw-degrading microbial communities [18].

There are numerous microorganisms in nature that can grow in low-temperature conditions, which are now the focus of many studies. For example, Boboua et al. (2023) suggest that the low-temperature-resistant microbial consortium LTF-27 is the most hydrolytic and acidifying bacterial consortium for corn straw degradation, with the effects of hydrolytic substrate concentration, initial pH, and inoculum quantity investigated using single-factor testing and response surface methodology [19]. Another case has been reported based on *Bacillus cereus* strain W118, which demonstrated effective rice straw degradation at temperatures as low as 4 °C, showing high potential for straw degradation in a low temperature environment [20]. Another low-temperature, high-efficiency cellulose-degrading bacterium (*Bacillus subtilis* K1) was detected with endo-β-glucanase, exo-β-glucanase, and β-glucosaccharase activities at 10 °C [14].

It is expected that the cold-adapted microorganisms have a diverse set of answers to the numerous issues that arise from surviving in cold conditions. Even though small layers of liquid water persist in frozen soils, water supply is a critical concern for life [21,22]. In addition, microbes must contend with decreased thermal energy, which delays the passage of nutrients and wastes, under conditions with an osmotic imbalance, which can also result in cell structure damage [23,24]. Other sources of direct physical harm to microbial cells are ice crystals that form both inside and outside of the cells [25]. Furthermore, the formation of ice crystals can also lead to the rupture of cell membranes, causing the leakage of intracellular components and ultimately resulting in cell death. In these cold-adapted microorganisms, there is an upregulation of membrane transport proteins under cold conditions that is necessary to overcome the hyperosmotic stress, improve nutrient diffusivity, and facilitate cellular waste disposal [26]. 

Using a genomic approach, researchers discovered that cold-adapted microorganisms have an overrepresentation of genes involved in cell membrane synthesis, as well as a downregulation of genes encoding outer membrane structures such as flagella, chemotaxis proteins, and iron uptake receptors [27,28]. These findings suggest that cold-adapted microorganisms have adapted to their environment by altering their membrane composition and reducing energy expenditure on extracellular structures. In order to combat desiccation, osmotic imbalance, and ice crystals, microorganisms accumulate solutes such as glycine, betaine, sucrose, and mannitol, among others [29]. Another substance psychrophiles use is exopolysaccharide, which lowers the freezing point and ice nucleation temperature of water and helps psychrophiles trap water, nutrients, and metal ions [30]. It also plays a role in cellular aggregation, biofilm formation, and the protection of extracellular enzymes [31,32]. Trehalose disaccharide is another chemical that works well in cold settings to prevent protein denaturation and aggregation, cell membrane stability, and free radical annihilation [33]. They also synthesize cold-adapted enzymes with higher activity at low temperatures and a more flexible structure, antifreeze proteins bind to ice crystals, damping their damage to cellular structures, cold acclimation proteins, and cold shock response proteins [34,35].

In the complex network of soil communities, fungi and bacteria can work together to degrade cellulose [36]. To access the cellulose, it is necessary to degrade the lignin matrix around it. Although several bacterial species can degrade lignin, usually fungi are better adapted for this process [37]. Fungal and bacterial species appear to prefer different substrates, but most of their interactions appear to be mutually beneficial [2]. A mixture of microorganisms is more effective in the degradation of complex plant residue materials than a single microbial strain [38]. In addition, richer and more functionally diverse microbial communities are better equipped to degrade lignocellulosic agricultural residues, and the richer the community becomes, the stronger its ability to degrade the straw residue [39]. In mixed cultures, symbioses between cellulose-degrading organisms and those that lack this ability can also increase cellulose degradation compared with single-strain cultures [40]. Moreover, some microorganisms can only grow in mixed cultures and depend on this condition to survive [41,42]. However, even with today’s technology, understanding the extent of the relationship between the two groups remains difficult.

## 2. Materials and Methods

### 2.1. Isolation of Microorganisms

The microbial consortium was isolated from a soil sample obtained from the Panshi City area in Jilin Province, China (43°8′41.75″ N 126°5′44.99″ E) based on the following procedures. First, a 5 g of soil sample was mixed with 100 mL of tryptic soy broth and agitated at 200 rpm and 28 °C for 30 min. Then, 1 mL of sample was transferred to 100 mL of new medium and maintained for 48 h under the same conditions. Finally, 1 mL of this culture was transferred to 100 mL of Hutchinson medium; 1 L of Hutchinson medium contained the following ingredients: 1.0 g of KH_2_PO_4_, 2.5 g of NaNO_3_, 0.1 g of NaCl, 0.1 g of CaCl_2_, 0.3 g of MgSO_4_·7H_2_O, and 0.01 g of FeCl_3_ (pH 7.2–7.3), using three strips of filter paper (1 cm × 10 cm) as the carbon source. The cultures were maintained at 28 °C and 80 rpm. Every 4 d, the 5 mL sample was transferred to fresh medium, and the temperature was decreased by increments of 2 °C until 15 °C. Each generation of the microorganisms was checked for filter paper degradation, and cultures showing no degradation were discarded. The degradation effect was evaluated according to the degree of damage of the filter paper. Specifically, the level of degradation was ranked to four classes, i.e., the expansion the filter paper edge was observed (+), the filter paper expanded and bended (++), the shape of filter paper became irregular (+++), and the filter paper became mushy (++++). After a stable consortium was established, this process was replicated over 10 generations to ensure that the cellulose-degrading capability was maintained over time.

### 2.2. Determination of Straw Weight Loss Rate

For this experiment, 2 g of corn straw was collected from the Experimental Corn Field at Jilin Agricultural University, simmered, and dried in the oven for 24 h at 80 °C, and then mixed with 100 mL of cellulase production medium, which contained (NH_4_)_2_SO_4_ 3 g, MgSO_4_ 0.4 g, CaCl_2_ 0.4 g, KH_2_PO_4_ 1 g, Tween-80 1 mL, yeast extract powder 0.5 g, peptone 2 g, and ultrapure water 1000 mL. Then, 10 mL of the microorganism culture was inoculated into cellulase production medium. The mixture was allowed to degrade cellulose for 24 d at 15 °C and 80 rpm. Every 2 d, a sample was taken, the corn straw was filtered, dried in the oven for 24 h, and weighed. Each experiment was repeated with three biological replicates.

### 2.3. Microscopic Analysis

Corn straw samples were collected during the degradation process described above after 3, 14, and 24 d of incubation and dried in the oven for 24 h at 80 °C. The samples were observed and photographed using an XL-30 ESEM FEG scanning electron microscope (FEI Company, Hillsboro, OR, USA).

### 2.4. Sequencing and Gene Annotation Analysis

The liquid bacterial culture, which had been collected for the determination of straw weight loss, was also sampled at 3, 15, and 24 d and sent to Personal Medicine (Shanghai, China) for bacterial 16S rRNA and fungal 18S rRNA sequencing. PCR amplification of the bacterial 16S rRNA genes V3–V4 region was performed using primers 338F (5′-ACTCCTACGGGAGGCAGCA-3′) and 806R (5′-GGACTACHVGGGTWTCTAAT-3′), and primers ITS5 (GGAAGTAAAAGTCGTAACAAGG) and ITS2 (GCTGCGTTCTTCATCGATGC) were used to amplify the 18S rRNA of fungal taxa. The samples were sequenced using Illumina high-throughput paired-end (2 × 250 bp) sequencing using a NovaSeq 6000 SP Reagent kit (500 cycles; Personal Medicine, Shanghai, China). VSEARCH was used for depriming, splicing, quality filtering, deduplication, chimera removal, and clustering. The resulting operational taxonomic units (OTUs) were annotated using QIIME2. The Greengenes (Release 13.8) and Silva (Release 138) databases were used for obtaining the sequences of the 16S rRNA genes of bacteria or archaea. For eukaryotes, the NCBI nt, UNITE (Release 8.0), and Silva (Release 132) databases were used. For the feature sequences of each amplicon sequence variant or the representative sequences of each NAïve, the QIIME2 species annotation was performed using a pre-trainnaïveive Bayesian classifier with default parameters. For the nt or nr database, the BROCC algorithm was used. In short, BLASTn or BLASTx was used first, and then the sequence was compared with the nucleic acid or protein sequence in the nt or nr databases, respectively. Finally, the brocc.py script was applied to obtain annotation information based on the recommended parameters. The alpha and beta diversities of the resulting OTUs were analyzed using QIIME2 (2019.4) and R, and the ggplot2 package was used for visualization. The metabolic pathway prediction was conducted using PICRUSt2 according to the bacterial 16S rRNA of bacterial and fungal 18S rRNA sequences. The databases MetaCyc (https://metacyc.org/; accessed on 5 January 2023), KEGG (https://www.kegg.jp/; accessed on 5 January 2023), and COG (https://www.ncbi.nlm.nih.gov/COG/; accessed on 5 January 2023) were used to map the sequences based on samples of 3, 15, and 24 d. Data analysis was performed using the genescloud platform (https://www.genescloud.cn/; accessed on 5 January 2023) with eukaryotes and prokaryotes analyzed separately.

Ranking analysis such as principal coordinate analysis (PCoA) are only exploratory methods, but not statistical tests. Therefore, both the distribution law presented in the ranking diagram and the grouping rules presented in cluster analysis were verified. One commonly used verification method is the permutational multivariate analysis of variance (PERMANOVA), which is a multivariate analysis of variance based on permutation tests, assuming that samples within groups are more similar than samples between groups [43]. In other words, it tests whether the within-sample distance for a group is different from the between-group sample distance. In our study, the adonis function or adnois2 function of the R language vegan package was used to perform this calculation.

After obtaining the abundance data of metabolic pathways, the metabolic pathways with significant differences between groups were further explored. Here, we used the method of metagenomeSe of the R software (4.0.3) script. First, the unflattened ASV/OTU table was used, and then the upregulated group and the control group were selected, following the tutorial example for metagenomeSeq. A call to the fitFeatureModel function was used and the zero-inflated log-normal model was required to fit the distribution of each ASV/OTU, and the significance of the differences was determined based on the fit results of the model.

## 3. Results

### 3.1. Cellulose Degradation Situation

**Figure 1:**
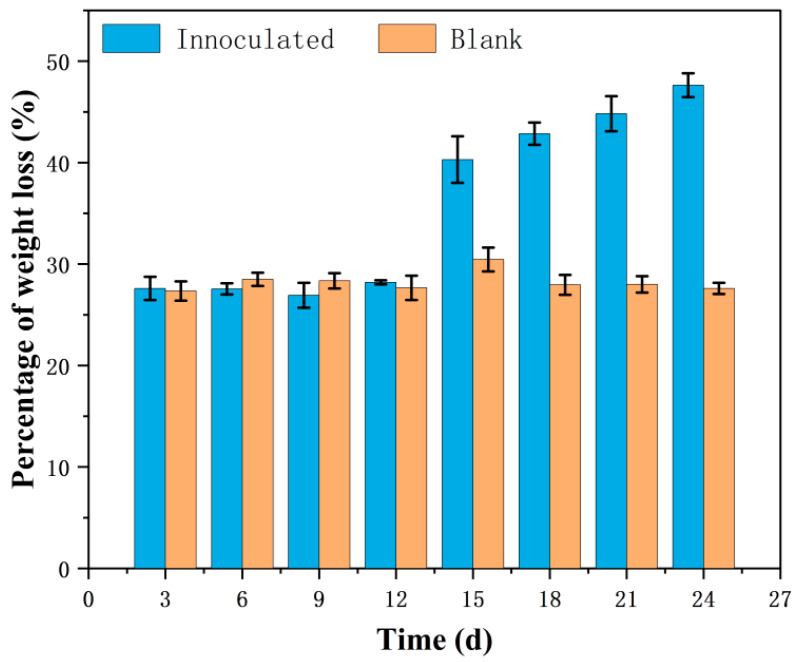
Percentage of corn straw weight loss over incubation time.

Compared with the uninoculated blank, the isolated microbial consortium was able to completely degrade almost all the filter paper in 6 d of incubation. The results for the corn straw weight loss rate showed that in 24 d of incubation, the corn straw weight was reduced by 48% compared with the initial weight (Figure 1). The blank, uninoculated samples remained stable with a weight loss of about 28%, which could be attributed to sample manipulation and the mechanical breakage of the corn straw fibers caused by rotation in the incubator. This tendency to lose corn straw weight due to degradation began to become slow from day 6 after inoculation and was accelerated over time, showing evident variations during 15 d of incubation.

**Figure 2:**
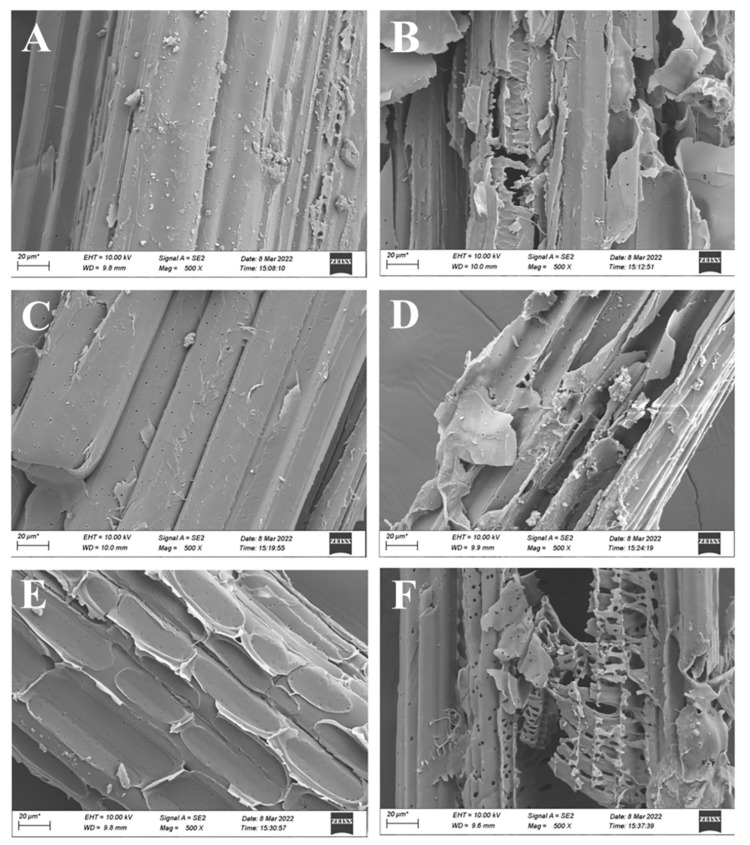
Scanning electron microscope images showing damage to the corn straw fibers during treatment without (blank) and with the isolated microbial consortium in 3 d (**A**,**B**), 15 d (**C**,**D**), and 24 d (**E**,**F**), respectively.

Microscopic analysis of the corn straw fibers revealed structural changes in the corn straw during incubation, showing the degradation capabilities of the microbial consortium. The results showed that corn straw treated with the isolated microbial consortium presented with more damaged fibers (Figure 2B,D,F) than the corn straw samples that were maintained in the same conditions but not exposed to the microorganisms (Figure 2A,C,E), and even a brief exposure to the microbial consortium caused scarring and tearing on the surface of the corn straw fibers (Figure 2A,B). Prolonged exposure to this microbial consortium aggravated the cracking and breaking of the fiber surfaces (Figure 2B,D,F).

### 3.2. Microbial Succession

**Figure 3:**
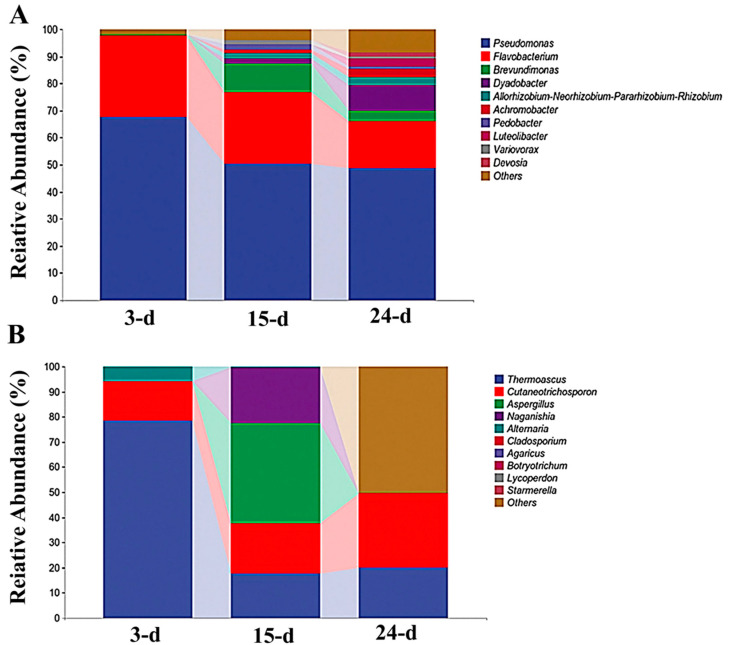
Relative abundance of bacterial (**A**) and fungal (**B**) genera over time.

The composition of genera in the community changed through time (Figure 3A). However, *Pseudomonas* and *Flavobacterium* were present in the majority of the samples, and their relative abundance decreased over time, while other genera, such as *Dyaodobacter,* became more prevalent. *Brevundimonas* appeared to spike in relative abundance at 15 d but had decreased by 24 d. Over time, the fungal genera were less consistent. The majority of fungi in the 3-day sample were identified as *Thermoascus*, which showed significantly decreased relative abundance over time. The relative abundance of *Cutaneotrichosporon* was relatively constant in all samples, although a slight increase occurred at the end of the incubation period. *Alternaria* was clearly relatively abundant in the 3-day sample, but this genus decreased over time. There were spikes in the relative abundances of *Naganishia* and *Aspergillus* in the 15-day samples. However, these genera largely disappeared by day 24 of incubation (Figure 3B).

**Figure 4:**
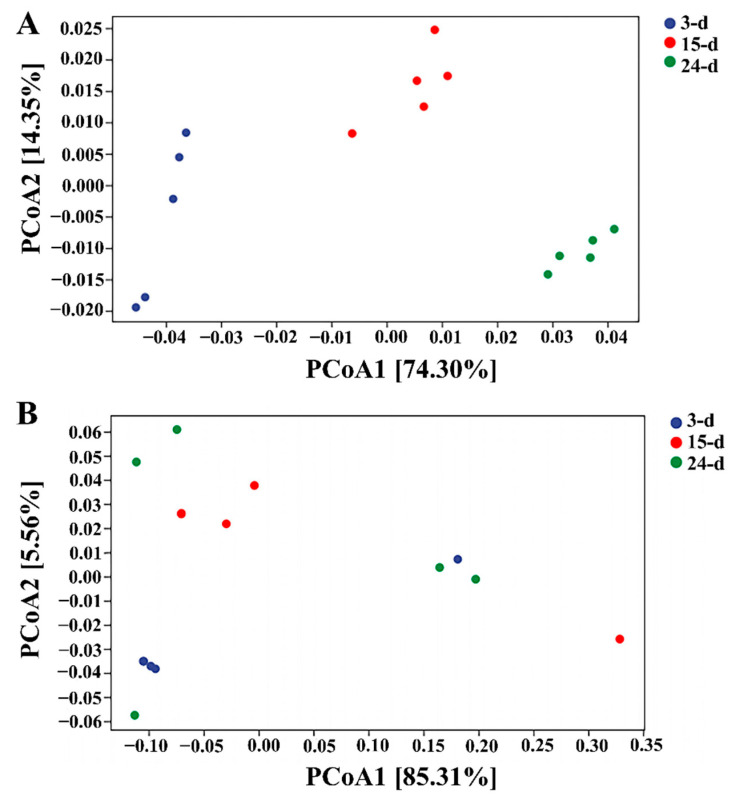
Functional principal coordinate analysis (PCoA) for bacterial (**A**) and fungal (**B**) samples. The 3-, 15-, and 24-day samples are shown in blue, red, and green, respectively.

Based on the species annotation and relative abundance at the genus level, the 35 most relatively abundant genera were identified and clustered within the sample groups to generate a heatmap. For bacteria, the most related samples were identified in the 3- and 15-day samples. In the 3-day samples, the most relatively abundant genera were *Pseudomonas* and *Herminiimonas*. In the 15-day samples, *Brevundimonas*, *Pedobacter*, and *Variovorax* were the most relatively abundant genera. Finally, in the 24-day samples, the most relatively abundant genera were *Devosia*, *Mesorhizobium*, *Luteolibacter*, *Pseudoxanthomonas*, *Achromobacter*, *Sphingomonas*, *E1B-B3-114*, *Dyadobacter*, *Cellvibrio*, and *Sphingopyxis* (Figure 4A). For fungi, the 3- and 24-day samples were the most closely related. *Thermoascus*, *Alternaria*, *Starmerella*, *Trametes*, and *Debaryomyces* were the most relatively abundant genera at 3 d. At 15 d, the most relatively abundant genera were *Vishniacozyma*, *Pichia*, *Gibberella*, *Lycoperdon*, *Didymella*, *Aspergillus*, and *Naganishia*. In the 24-day samples, the most relatively abundant genera were *Cladosporium*, *Metarhizium*, *Papiliotrema*, *Cutaneotrichosporon*, and *Solicoccozyma* (Figure 4B).

### 3.3. Diversity Analysis

Alpha diversity measured the diversity of species and their abundance in each sample. The following indexes of alpha diversity were used: the observed species, Shannon, Simpson, and Chao1 indexes. Bacterial and fungal samples were analyzed separately. In bacteria, a total of 542, 205, and 145 reads in samples collected at 3, 15, and 24 d were classified as OTUs, respectively, with a total of 18, 28, and 34 OTUs identified at genus level, and a total of 108, 137, and 159 OTUs identified at species level, respectively. In fungal populations, a total of 4021, 3922, and 3875 reads in samples collected at 3, 15, and 24 d were classified as OTUs, respectively, with a total of 2, 2, and 2 OTUs identified at genus level, and a total of 13, 13, and 13 OTUs identified at species level, respectively. Table 1 showed the alpha diversity indexes for the bacterial samples taken at three time points of the experiment (3, 15, and 24 d), respectively. For the bacteria present in the consortium isolated, all measures of alpha diversity clearly showed that diversity and abundance of species increased with treatment time. For the fungal samples (Table 1), the alpha diversity appeared to be maintained over time, with the samples at 24 d having slightly larger numbers of observed species (Chao1 and Shannon indexes); for the Simpson index, a larger number of species was indicated in the 15-day sample. For both the bacterial and fungal samples, the observed species curve began to plateau after 5000 sequences (Figures S1 and S2), and all had a coverage greater than 0.99, indicating good coverage of the species present in the samples.

**Figure 5:**
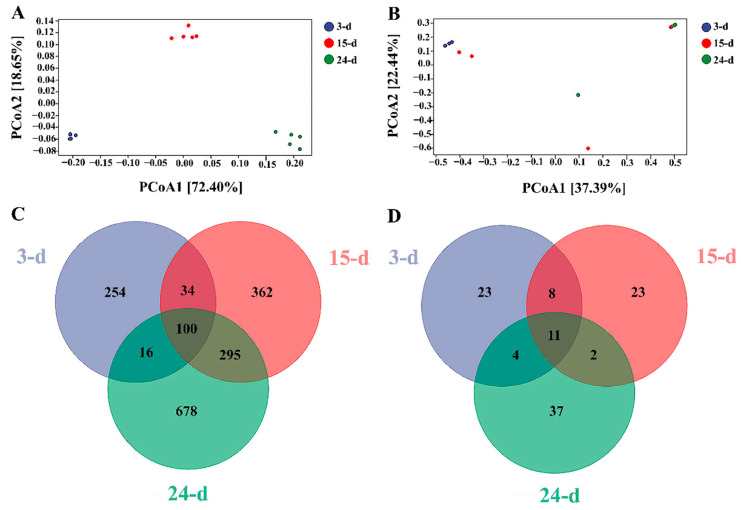
Changes in bacterial and fungal diversity over time, showing the beta diversity based on principal coordinate analysis (PCoA) of bacteria (**A**) and fungi (**B**) present in the isolated microbial consortium, as well as the variations in the number of bacterial (**C**) and fungal (**D**) OTUs over time. The 3-, 15-, and 24-day samples are shown in blue, red, and green, respectively.

While alpha diversity measured the variance within each sample, beta diversity can determine whether there were differences between samples. For the bacterial samples, the results of PCoA indicated that samples from each time point differed from the others, with the 3- and 24-day samples being the most different from one another (Figure 5A). For fungal samples within the isolated microorganism consortium, the results of PCoA showed that the samples taken after 3 d of incubation and those collected at 24 d differed the most, except for one of the repetitions after 3 d of incubation that seemed to be similar to that at 24 d (Figure 3B). The samples at 15 d showed similarities with both the 3- and 24-day samples. A Venn diagram was used to describe the distribution of the OTUs at species level in the different samples, showing an increase in the numbers of OTUs over time for both the bacterial and fungal samples. For bacteria, the exclusive number of OTUs at each time point was increased from 254 in the 3-day samples to 362 in the 15-day samples and finally to 678 in the 24-day samples (Figure 5C). The exclusive number of OTUs in fungal samples remained constant at 23 for the 3- and 15-day samples but was increased to 37 for the 24-day samples (Figure 5D). The core microbiome was composed of 11 OTUs.

The beta diversity was an indicator for describing the difference between groups, specifically the timeframe of data collection. The PCoA revealed that each bacterial sample was unique from the others, demonstrating that the bacterial community existed as three distinct populations throughout the trial. For fungal samples, the fungal communities mostly acted as a single population over time, except at the beginning and conclusion of the trial. The Venn diagram also showed the slow changes in the fungal community compared with the bacterial populations. This difference was probably due to fungal development and adaptation being slower than those for bacteria, as the number of fungal ASV/OUT difference between samples was only 14 between the beginning and conclusion of the trial but was stabilized for most of the experimental time, whereas bacterial ASV/OUT varied more dramatically between samples at each stage of the trial.

**Figure 6:**
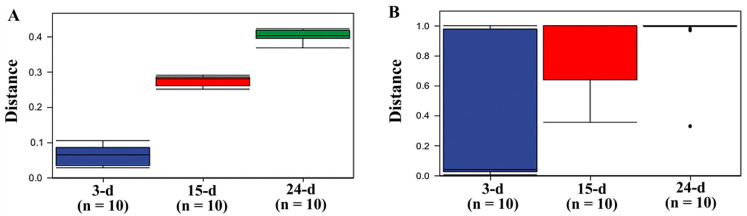
PERMANOVA for bacterial (**A**) and fungal populations (**B**) based on 3-, 15-, and 24-d samples.

To verify this speculation, PERMANOVA was carried out to calculate if samples within groups were more similar to samples between groups or to each other, determining whether the within-sample distance for a group differed from the sample distance between groups. The results clearly showed that bacteria behaved as three different populations at each sample time (Figure 6), while no clear distinction was detected between the three sample times, with all samples overlapping with the others. Even though the first and last samples showed the greatest difference, the fungal population could still be considered a single population throughout the experimental time.

### 3.4. Functional Principal Component Analysis

**Figure 7:**
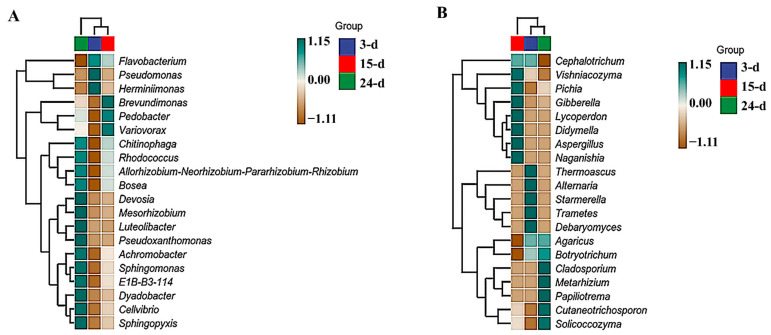
Relative abundance heatmap for bacterial (**A**) and fungal (**B**) samples at genus level. The 3-, 15-, and 24-day samples are shown in blue, red, and green, respectively.

Functional unit principal component analysis showed that all the repetitions from each sample were distinct, revealing functional diversity over time for bacteria, while no clear distinction was detected in fungi between samples at different times and no cluster of samples was formed (Figure 7). These results indicated that the bacterial samples were functionally working as three distinctive populations depending on the sample time. In contrast, all fungal samples were functionally similar and it was not possible to distinguish them as separate functional communities.

### 3.5. Metabolic Pathways in Bacteria and Fungi

**Figure 8:**
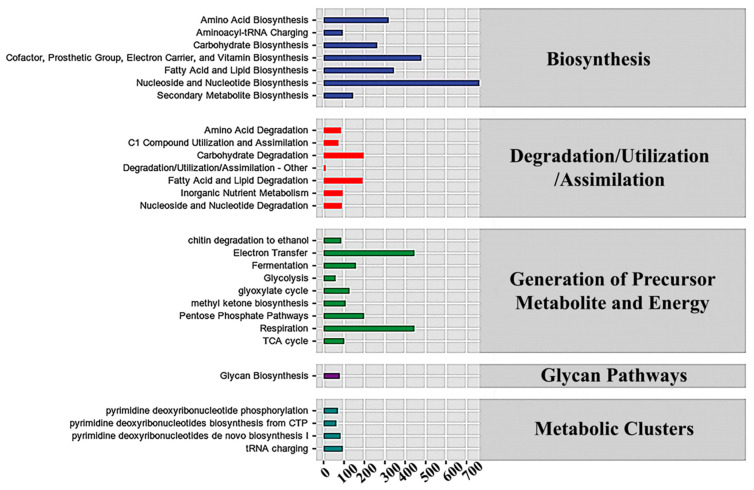
Metabolic pathways for bacterial population. The abscissa represents the abundance (unit: per million KO/PWY/COG) or counts of functional pathways/classifications, the ordinates are the functional pathways/classifications of the second classification level of KEGG/MetaCyc/COG, and the far right presents the first level pathway/class to which this pathway belongs.

**Figure 9:**
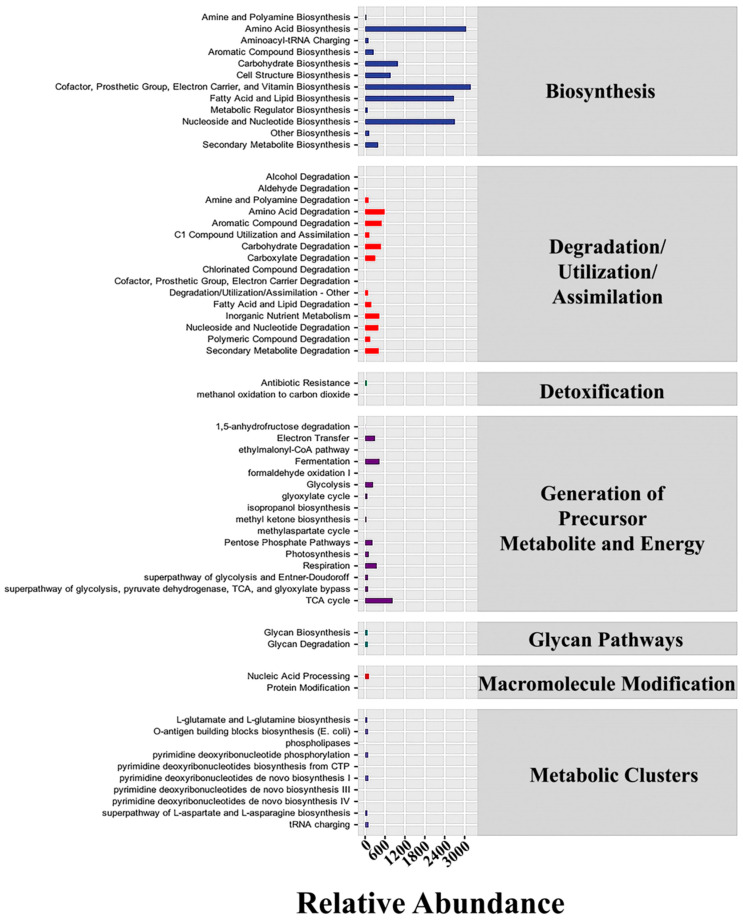
Metabolic pathways for fungal population. The abscissa is the abundance (unit: per million KO/PWY/COG) or counts of functional pathways/classifications, the ordinates are the functional pathways/classifications of the second classification level of KEGG/MetaCyc/COG, and the far right is the first level pathway/class to which this pathway belongs.

The abundance value of the metabolic pathway for the obtained functional unit was determined using metabolic pathway databases, including the KEGG, MetaCyc, and COG databases. The results for bacterial populations revealed an intense concentration of genes for membrane components, including lipid biosyntesis and electron carriers and other membrane transporters, as anticipated in cold-adapted bacteria (Figure 8 and Table S1). The Cofactor, Prosthetic Group, Electron Carrier, and Vitamin Biosynthesis, and the Amino Acid, Nucleoside and Nucleotide, and Fatty Acid and Lipid Biosynthesis were the most prominent pathways in bacteria. These results were consistent with those expected for cold-adapted bacteria adjusting to survive in cold conditions. Similarly, in fungal populations, genes involved in the Nucleoside and Nucleotide, Cofactor, Prosthetic Group, Electron Carrier, and Vitamin Biosynthesis, and the Electron Transfer, Respiration and Fatty Acid, and Lipid Biosynthesis were the most abundant (Figure 9 and Table S2).

In addition, there were a large number of genes involved in the breakdown of substances subcategory, e.g., amino acid decomposition and carbohydrate degradation. It was noted that bacteria exhibited about 4806 genes involved in carbohydrate degradation. Additionally, the fungal analysis showed a high use of genes involved in carbohydrate breakdown, lipid and fatty acid decomposition, and biosynthesis of various cell components, as well as a large number of genes involved in energy utilization. Fungal samples were revealed to have about 1972 genes involved in carbohydrate degradation. To make the comparison fairer, the percentage of the total genes was used in these comparisons between bacterial and fungal populations. The carbohydrate decomposition genes in bacteria accounted for 2.20% of their overall gene frequency. In contrast, the proportion for fungi was 3.73%. Although the percentage was a little higher for fungi, the assumption that fungi were the main factor in the decomposition of corn straw needed to be verified. Also, the percentage for bacteria may have been low, whereas in reality the genetic diversity of bacterial population is generally higher than that of the fugal samples. Further investigations are necessary to verify the findings revealed in this study.

## 4. Discussion

In this work, we isolated a low-temperature-adapted microorganism consortium for corn straw degradation using the filter paper degradation approach. This consortium was stable and maintained its degradation ability over time. The ability to degrade lignocellulosic waste materials was confirmed by the weight loss of corn straw over time, which was significantly higher than the control without microorganisms under the same conditions. Scanning electron microscopy was used to confirm the damage to the corn straw fibers caused by the microorganisms. This damage was limited, even though it was apparent after only 3 d of exposure to the microorganism consortium.

The increase in alpha diversity over time for the bacterial and fungal samples indicated an increase in the diversity of the microbial taxa found in the isolated microorganism consortium. Bacteria and fungi have very distinct behaviors in their diversity changes through time. On one hand, bacteria in the community behaved as essentially three distinct communities depending on the sampling time, not only in their species diversity but also in their functionality. On the other hand, the changes in fungal species diversity did not show a clear progression, neither in diversity nor in function. Therefore, fungi could be considered a single community over the course of the entire experiment.

At the beginning of the experiment, the bacterial genus *Pseudomonas* and the fungal genus *Thermoascus* were prominent. These two genera are well known for their ability to degrade cellulose and withstand low temperatures. *Thermoascus* exhibits a variety of amylase, cellulase, pectinase, and xylanase activities [1], and it has also been reported as a cellulose-degrading fungus at high temperatures [44]. *Cutaneotrichosporon curvatus* has been reported to be useful in the production of sustainable single cell oil from undetoxified lignocellulosic hydrolysates [45,46]. The genus *Aspergillus* contains some well-known members capable of degrading cellulose and using other plant materials as a source for alpha-glucuronidase [47,48]. *Naganishia diffluens* is another well-documented fungal taxon with cellulose degradation capabilities that has been investigated to explore its enzymatic production potential [49,50]. Previous studies showed that *Pseudomonas* was a dominant genus in low-temperature cellulose degradation, and a key component in many other communities of soil cellulose-degrading microorganisms [51,52]. Furthermore, this genus is known for its cold-adaptation capabilities and can be a source of many cold-adapted enzymes for bioprospecting [53]. Moreover, *P. fluorescens*, a recognized cold-adapted bacterium, can cause food spoiling in a variety of frozen items while often being unchecked in soil or water [54]. *Pseudomonas stutzeri* has been identified as a bioremediation agent that accelerates cellulose breakdown and agrochemical biodegradation [55,56]. Last but not least, *P. putida* is an effective and well-known plant material degrader [56,57]. These findings highlight the potential of *Pseudomonas* species as bioremediation agents for different environmental contaminants. Further research is needed to explore their full capabilities and optimize their use in bioremediation strategies. There is no doubt that these are only just a few of the actors in the degradation process. For example, a recent study showed that *Cellvibrio japonicus* was identified as having cellulose degradation activity and a source of new cellulase [58,59]. This implies that investigating the genetic potential of *C. japonicus* may result in the identification and development of novel, environmentally friendly techniques for cellulose breakdown, which are crucial for tackling environmental issues, which include waste management and the manufacture of biofuels. *Sphingomonas*, also prominently displayed in the multilevel species composition map in our study, has already been extensively investigated in cellulose degradation as well as other agricultural composting systems [60,61]. *Brevundimonas naejangsanensis* is credited with having excellent bioremediation capabilities for dicarboximide fungicides in brunisolic soil, which are used to prevent serious plant infestations. Therefore, the potential of *B. naejangsanensis* to remediate dicarboximide fungicides and other xenobiotics from soil may have significant implications for the full bioremediation capabilities of this microbial consortium.

In general, the most crucial members of the community can be excellent bioremediation agents that might support the breakdown of lignocellulolytic material and the restoration of soil health. This could make the microbial consortium isolated not only a remedy for the problem of corn straw degradation, but also a significantly potent agent in general soil health recovery that may be useful in many agroindustrial processes. This could lead to a more sustainable and ecofriendly approach for waste management and soil health improvement. Additional research is required to fully understand the mechanisms regulating the microorganisms in the consortium.

The bacterial diversity was richer than that of the fungal diversity and showed a clear and distinctive succession of microorganisms over time. The fungal diversity was more erratic, and the only noticeable difference was between the beginning and end of the incubation period. This suggested that while *Pseudomonas* may play a dominant role in the degradation of cellulose in soil environments, it is not the sole microorganism involved in the process. The results of PERMANOVA revealed that while the fungal population was stable and consistent throughout the entire experiment, the bacterial population was more akin to three different populations, each representing one of the three sample times. This indicates a rapid change and evolution in the members of the bacterial community, which adjust themselves as the environmental conditions shift due to the increased degradation of corn straw during the experiment.

For fungal populations, a clear distinction was not detected by the functional unit PCoA between the different sampling times. Although some of the repetitions from the 15- and 24-day samples were grouped and some of the repetitions of the 3- and 24-day samples were grouped, they were inconsistent across all sample repetitions. While bacteria behaved as three functionally separate populations during the three sampling times, it is possible that many of the genera that gained relative dominance in the 15- and 24-day samples were in reality “cheating” genera that took advantage of the nutrients made available by *Thermoascus* and *Pseudomonas*. More investigation is needed to distinguish “cheating” microorganisms from real cellulose-degrading microorganisms. For example, *Dyadobacter*, a bacterial genus that is one of the significant contributors to the glycolytic pathway, has been reported as heterotrophic-nitrifying but not as cellulose-degrading bacteria [62]. Thus, it is possible that *Dyadobacter* and other microorganisms may take advantage of the increased nutrient availability as the corn straw is degraded. To explicitly determine whether the microorganisms that gained dominance were true degraders of cellulose, further investigation is needed. It is important to consider that the balance of nutrients and metabolites in the community may be important to maintain the degradation of cellulose, hemicellulose, and lignin. As a result, it is impossible to label a microorganism as “cheating” at this point because it may be performing other, indirect cellulose-degrading functions that could be critical to the community’s long-term survival.

As expected in cold-adapted microbes, both bacterial and fungal groups showed an abundance of genes for membrane components. Furthermore, the decomposition of compounds subcategory contains a significant number of genes in both bacterial and fungal samples. Amino acid breakdown and carbohydrate degradation seem to be the most important processes for bacteria, while the fungal analysis indicated that genes were extensively involved in energy use, carbohydrate degradation, lipid and fatty acid decomposition, and biosynthesis of different cell components. Bacterial carbohydrate decomposition genes accounted for 2.20% of their overall gene frequency. In comparison, the proportion for fungi was 3.73%. Although the proportion of fungi is slightly greater, it is premature to assume that the fungi are the primary factors in the decomposition of straw. Also, while the proportion of bacteria may be low, the bacterial genetic variety is greater than that in fugal samples, suggesting that there are probably other factors involved in this process. Additionally, the interactions between the groups may be vital to maintain high efficiency of degradation.

The functional relationships between bacteria and fungi in the consortium are still uncertain. Although the bacteria showed clear progression and succession, the trajectories of the fungal samples are more difficult to predict and do not follow a fixed pattern. Therefore, additional experiments will be required to determine the nature of their relationship, if any, in the consortium. Procaryotes have a high level of adaptability and can quickly evolve to fit their environment. In the fungal samples, we observed more conserved gene content, with similar genes shared across different fungal taxa, suggesting that eucaryotes have a stable and well-conserved genetic makeup that enables them to maintain their distinctive features and functions.

Overall, we are aware that procaryotes and eucaryotes have different strategies to maintain their genetic diversity and stability, which reflect their distinct lifestyles and ecological roles. However, these differences do not necessarily mean that these organisms are isolated from each other, as they often interact and coexist in various ecological niches, especially in soil communities, where they work closely together, often generating mutually beneficial relationships or fierce competition. Microbial diversity and its ecological context are essential for several important applications. For example, the understanding of soil systems may lead to more sustainable agriculture practices. Furthermore, studying microbial communities is necessary for interpreting natural ecosystems, as well as developing solutions to address societal issues related to human health and environmental sustainability.

## 5. Conclusions

We successfully identified a microbial community capable of degrading corn straw at low temperatures. The microorganisms isolated showed fast cellulose-degrading capabilities, as confirmed by scanning electron microscopy and the weight loss in corn straw. Bacterial carbohydrate decomposition genes accounted for 2.20% of their overall gene frequency. In comparison, the proportion for fungi was 3.73%. While the proportion of bacteria may be low, the genetic variety is greater than that in fungal samples. The bacterial genus *Pseudomonas* and the fungal genus *Thermoascus* played prominent roles in the group. Both genera have previously been reported to be important in other lignocellulose waste-degrading microbial consortia. Bacteria and fungi identified in the consortium showed high abundance of genes for membrane components, with amino acid breakdown and carbohydrate degradation being the most important for bacteria, while fungi have more genes involved in energy use, carbohydrate degradation, lipid and fatty acid decomposition, and biosynthesis.

## Figures and Tables

**Table 1:** Coverage and indexes of alpha diversity for bacteria and fungi included in the isolated microbial consortium.

Sample Type	Sample Time	Coverage	Chao1	Observed	Shannon	Simpson
Bacteria	3 days	0.999	164.28	151.34	2.074	0.685
	15 days	0.998	392.01	352.52	3.523	0.811
	24 days	0.997	560.31	500.02	4.476	0.875
Fungi	3 days	0.999	17.06	16.88	0.512	0.163
	15 days	0.999	16.83	16.8	0.923	0.33
	24 days	0.999	17.32	17.22	1.022	0.322

## Data Availability

Data are contained within the article and Supplementary Materials.

## Supplementary Materials

The following supporting information can be downloaded at: https://www.mdpi.com/article/10.3390/microorganisms12061059/s1, Table S1: Abundance of metabolic pathways (levels 1 and 2) identified in bacteria; Table S2: Abundance of metabolic pathways (levels 1 and 2) identified in fungi; Figure S1: Bacterial rarefaction curves; Figure S2: Fungal rarefaction curves.
