# Supplementary material for: Isolation and Characterization of a Low-Temperature, Cellulose-Degrading Microbial Consortium from Northeastern China
Source: Microorganisms. 2024 May 24;12(6):1059. doi: 10.3390/microorganisms12061059 (PMC11205951; doi:10.3390/microorganisms12061059)

Table S1. Abundance of metabolic pathways (levels 1 and 2) identified in bacteria.

Level 1	Level 2	Abundance	Percentage of total
Biosynthesis	Amine and Polyamine Biosynthesis	442.93	0.20%
Biosynthesis	Metabolic Regulator Biosynthesis	779.57	0.36%
Biosynthesis	Aminoacyl-tRNA Charging	1025.18	0.47%
Biosynthesis	Other Biosynthesis	1240.57	0.57%
Biosynthesis	Aromatic Compound Biosynthesis	2576.93	1.18%
Biosynthesis	Secondary Metabolite Biosynthesis	3955.75	1.81%
Biosynthesis	Cell Structure Biosynthesis	7715.58	3.53%
Biosynthesis	Carbohydrate Biosynthesis	9912.48	4.54%
Biosynthesis	Fatty Acid and Lipid Biosynthesis	26812.95	12.28%
Biosynthesis	Nucleoside and Nucleotide Biosynthesis	27097.9	12.42%
Biosynthesis	Amino Acid Biosynthesis	30465.06	13.96%
Biosynthesis	Cofactor, Prosthetic Group, Electron Carrier, and Vitamin Biosynthesis	31869.59	14.60%
Degradation/Utilization/Assimilation	Cofactor, Prosthetic Group, Electron Carrier Degradation	0	0.00%
Degradation/Utilization/Assimilation	Alcohol Degradation	7.93	0.00%
Degradation/Utilization/Assimilation	Chlorinated Compound Degradation	11.03	0.01%
Degradation/Utilization/Assimilation	Aldehyde Degradation	11.36	0.01%
Degradation/Utilization/Assimilation	Degradation/Utilization/Assimilation - Other	901.61	0.41%
Degradation/Utilization/Assimilation	Amine and Polyamine Degradation	1028.77	0.47%
Degradation/Utilization/Assimilation	C1 Compound Utilization and Assimilation	1271.64	0.58%
Degradation/Utilization/Assimilation	Polymeric Compound Degradation	1555.96	0.71%
Degradation/Utilization/Assimilation	Fatty Acid and Lipid Degradation	1863.2	0.85%
Degradation/Utilization/Assimilation	Carboxylate Degradation	3049.17	1.40%
Degradation/Utilization/Assimilation	Nucleoside and Nucleotide Degradation	4003.49	1.83%
Degradation/Utilization/Assimilation	Secondary Metabolite Degradation	4128.28	1.89%

Degradation/Utilization/Assimilation	Inorganic Nutrient Metabolism	4263.44	1.95%
Degradation/Utilization/Assimilation	Carbohydrate Degradation	4805.94	2.20%
Degradation/Utilization/Assimilation	Aromatic Compound Degradation	5013.74	2.30%
Degradation/Utilization/Assimilation	Amino Acid Degradation	5883.91	2.70%
Detoxification	Antibiotic Resistance	537.79	0.25%
Detoxification	methanol oxidation to carbon dioxide	3.78	0.00%
Generation of Precursor Metabolite and Energy	methylaspartate cycle	2.15	0.00%
Generation of Precursor Metabolite and Energy	formaldehyde oxidation I	2.42	0.00%
Generation of Precursor Metabolite and Energy	ethylmalonyl-CoA pathway	4.6	0.00%
Generation of Precursor Metabolite and Energy	isopropanol biosynthesis	15.23	0.01%
Generation of Precursor Metabolite and Energy	1,5-anhydrofructose degradation	38.56	0.02%
Generation of Precursor Metabolite and Energy	methyl ketone biosynthesis	448.85	0.21%
Generation of Precursor Metabolite and Energy	glyoxylate cycle	707.39	0.32%
Generation of Precursor Metabolite and Energy	superpathway of glycolysis and Entner-Doudoroff	887.4	0.41%

Generation of Precursor Metabolite and Energy	superpathway of glycolysis, pyruvate dehydrogenase, TCA, and glyoxylate bypass	916.03	0.42%
Generation of Precursor Metabolite and Energy	Photosynthesis	1149.4	0.53%
Generation of Precursor Metabolite and Energy	Pentose Phosphate Pathways	2253.92	1.03%
Generation of Precursor Metabolite and Energy	Glycolysis	2423.16	1.11%
Generation of Precursor Metabolite and Energy	Electron Transfer	2983.52	1.37%
Generation of Precursor Metabolite and Energy	Respiration	3534.41	1.62%
Generation of Precursor Metabolite and Energy	Fermentation	4332.19	1.98%
Generation of Precursor Metabolite and Energy	TCA cycle	8306.96	3.81%
Glycan Pathways	Protein Modification	732.78	0.34%
Glycan Pathways	Glycan Biosynthesis	819.13	0.38%
Macromolecule Modification	Glycan Degradation	1174.03	0.54%
Macromolecule Modification	Nucleic Acid Processing	0.02	0.00%
Metabolic Clusters	pyrimidine deoxyribonucleotides de novo biosynthesis IV	0.17	0.00%
Metabolic Clusters	phospholipases	0.23	0.00%
Metabolic Clusters	pyrimidine deoxyribonucleotides biosynthesis from CTP	0.24	0.00%

Metabolic Clusters	pyrimidine deoxyribonucleotides de novo biosynthesis III	7.55	0.00%
Metabolic Clusters	L-glutamate and L-glutamine biosynthesis	694.48	0.32%
Metabolic Clusters	superpathway of L-aspartate and L-asparagine biosynthesis	708.99	0.32%
Metabolic Clusters	O-antigen building blocks biosynthesis (E. coli)	919.23	0.42%
Metabolic Clusters	pyrimidine deoxyribonucleotide phosphorylation	949.37	0.43%
Metabolic Clusters	pyrimidine deoxyribonucleotides de novo biosynthesis I	992.24	0.45%
Metabolic Clusters	tRNA charging	1025.18	0.47%

Table S2. Abundance of metabolic pathways (levels 1 and 2) identified in fungi.

Level 1	Level 2	Abundance	Percentage of total
Biosynthesis	Amine and Polyamine Biosynthesis	442.93	0.20%
Biosynthesis	Metabolic Regulator Biosynthesis	779.57	0.36%
Biosynthesis	Aminoacyl-tRNA Charging	1025.18	0.47%
Biosynthesis	Other Biosynthesis	1240.57	0.57%
Biosynthesis	Aromatic Compound Biosynthesis	2576.93	1.18%
Biosynthesis	Secondary Metabolite Biosynthesis	3955.75	1.81%
Biosynthesis	Cell Structure Biosynthesis	7715.58	3.53%
Biosynthesis	Carbohydrate Biosynthesis	9912.48	4.54%
Biosynthesis	Fatty Acid and Lipid Biosynthesis	26812.95	12.28%
Biosynthesis	Nucleoside and Nucleotide Biosynthesis	27097.9	12.42%
Biosynthesis	Amino Acid Biosynthesis	30465.06	13.96%
Biosynthesis	Cofactor, Prosthetic Group, Electron Carrier, and Vitamin Biosynthesis	31869.59	14.60%
Degradation/Utilization/Assimilation	Cofactor, Prosthetic Group, Electron Carrier Degradation	0	0.00%
Degradation/Utilization/Assimilation	Alcohol Degradation	7.93	0.00%
Degradation/Utilization/Assimilation	Chlorinated Compound Degradation	11.03	0.01%
Degradation/Utilization/Assimilation	Aldehyde Degradation	11.36	0.01%

Degradation/Utilization/Assimilation	Degradation/Utilization/Assimilation - Other	901.61	0.41%
Degradation/Utilization/Assimilation	Amine and Polyamine Degradation	1028.77	0.47%
Degradation/Utilization/Assimilation	C1 Compound Utilization and Assimilation	1271.64	0.58%
Degradation/Utilization/Assimilation	Polymeric Compound Degradation	1555.96	0.71%
Degradation/Utilization/Assimilation	Fatty Acid and Lipid Degradation	1863.2	0.85%
Degradation/Utilization/Assimilation	Carboxylate Degradation	3049.17	1.40%
Degradation/Utilization/Assimilation	Nucleoside and Nucleotide Degradation	4003.49	1.83%
Degradation/Utilization/Assimilation	Secondary Metabolite Degradation	4128.28	1.89%
Degradation/Utilization/Assimilation	Inorganic Nutrient Metabolism	4263.44	1.95%
Degradation/Utilization/Assimilation	Carbohydrate Degradation	4805.94	2.20%
Degradation/Utilization/Assimilation	Aromatic Compound Degradation	5013.74	2.30%
Degradation/Utilization/Assimilation	Amino Acid Degradation	5883.91	2.70%
Detoxification	Antibiotic Resistance	537.79	0.25%
Detoxification	methanol oxidation to carbon dioxide	3.78	0.00%
Generation of Precursor Metabolite and Energy	methylaspartate cycle	2.15	0.00%
Generation of	formaldehyde oxidation I	2.42	0.00%

Precursor Metabolite and Energy			
Generation of Precursor Metabolite and Energy	ethylmalonyl-CoA pathway	4.6	0.00%
Generation of Precursor Metabolite and Energy	isopropanol biosynthesis	15.23	0.01%
Generation of Precursor Metabolite and Energy	1,5-anhydrofructose degradation	38.56	0.02%
Generation of Precursor Metabolite and Energy	methyl ketone biosynthesis	448.85	0.21%
Generation of Precursor Metabolite and Energy	glyoxylate cycle	707.39	0.32%
Generation of Precursor Metabolite and Energy	superpathway of glycolysis and Entner-Doudoroff	887.4	0.41%
Generation of Precursor Metabolite and Energy	superpathway of glycolysis, pyruvate dehydrogenase, TCA, and glyoxylate bypass	916.03	0.42%
Generation of Precursor Metabolite and Energy	Photosynthesis	1149.4	0.53%
Generation of Precursor Metabolite and Energy	Pentose Phosphate Pathways	2253.92	1.03%
Generation of Precursor Metabolite and Energy	Glycolysis	2423.16	1.11%
Generation of	Electron Transfer	2983.52	1.37%

Precursor Metabolite and Energy			
Generation of Precursor Metabolite and Energy	Respiration	3534.41	1.62%
Generation of Precursor Metabolite and Energy	Fermentation	4332.19	1.98%
Generation of Precursor Metabolite and Energy	TCA cycle	8306.96	3.81%
Macromolecule Modification	Protein Modification	0.02	0.00%
Glycan Pathways	Glycan Biosynthesis	732.78	0.34%
Glycan Pathways	Glycan Degradation	819.13	0.38%
Macromolecule Modification	Nucleic Acid Processing	1174.03	0.54%
Metabolic Clusters	pyrimidine deoxyribonucleotides de novo biosynthesis IV	0.17	0.00%
Metabolic Clusters	phospholipases	0.23	0.00%
Metabolic Clusters	pyrimidine deoxyribonucleotides biosynthesis from CTP	0.24	0.00%
Metabolic Clusters	pyrimidine deoxyribonucleotides de novo biosynthesis III	7.55	0.00%
Metabolic Clusters	L-glutamate and L-glutamine biosynthesis	694.48	0.32%
Metabolic Clusters	superpathway of L-aspartate and L-asparagine biosynthesis	708.99	0.32%
Metabolic Clusters	O-antigen building blocks biosynthesis (E. coli)	919.23	0.42%
Metabolic Clusters	pyrimidine deoxyribonucleotide phosphorylation	949.37	0.43%
Metabolic Clusters	pyrimidine deoxyribonucleotides de novo biosynthesis I	992.24	0.45%
Metabolic Clusters	tRNA charging	1025.18	0.47%

Figure S1. Bacterial rarefaction curves.

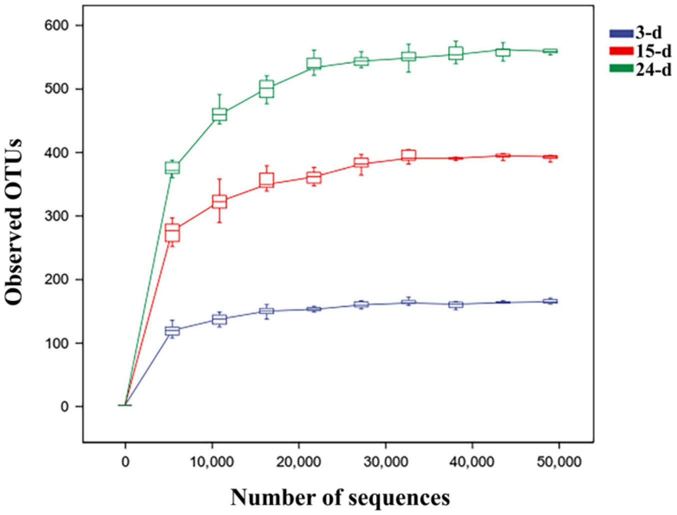

Figure S2. Fungal rarefaction curves.

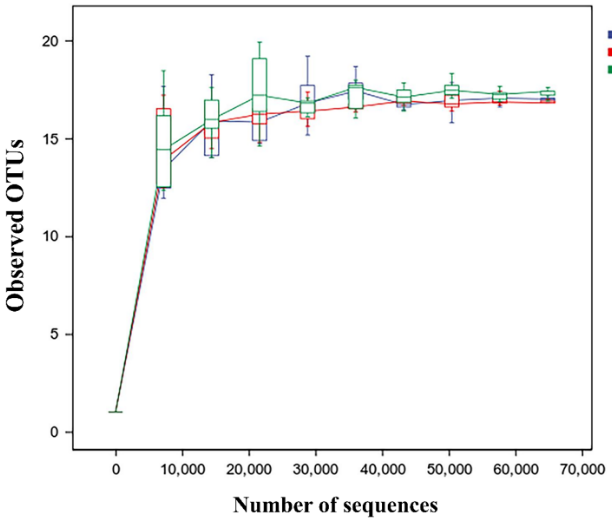

Supplement: Supplementary file 1 [file microorganisms-12-01059-s001.zip › microorganisms-3012345-supplementary.pdf]
